# Magnetocaloric Properties of Mn_1.1_Fe_0.9_P_0.5_As_0.5−*x*_Ge*_x_* (0 ≤ *x* ≤ 0.1) Compounds

**DOI:** 10.3390/ma10050529

**Published:** 2017-05-13

**Authors:** Patryk Wlodarczyk, Lukasz Hawelek, Maciej Kowalczyk, Malgorzata Kaminska, Marcin Polak, Andrzej Hudecki, Aleksandra Kolano-Burian

**Affiliations:** 1 Institute of Non-Ferrous Metals, ul. Sowinskiego 5, 44-100 Gliwice, Poland; lukaszh@imn.gliwice.pl (L.H.); malgorzata.kaminska@imn.gliwice.pl (M.K.); marcin.polak@imn.gliwice.pl (M.P.); andrzej.hudecki@gmail.com (A.H.); olak@imn.gliwice.pl (A.K.-B.); 2 Faculty of Materials Science and Engineering, Warsaw University of Technology, ul. Woloska 141, 02-507 Warsaw, Poland; mmkowalczyk@gmail.com

**Keywords:** magnetocaloric effect, adiabatic temperature change, crystal structure, intermetallic compound

## Abstract

Intermetallic compounds with the overall formula Mn_1.1_Fe_0.9_P_0.5_As_0.5−*x*_Ge*_x_* (*x* varies from 0 to 0.1) were investigated in order to study their magnetocaloric effect by monitoring the adiabatic temperature change, magnetic entropy change and their relation to structural parameters. It was found that the maximum of magnetocaloric effect was achieved for *x* = 0.02. Adiabatic temperature change for consolidated powder was equal to 2.75 K for the magnetic field change ΔB = 1.7 T for the sample with *x* = 0.02. For the pure non-doped sample, this parameter is much lower: ΔT_ad_ = 1.7 K @ ΔB = 1.7 T. This result was correlated with the change of structural parameters such as lattice constants and the unit cell volume.

## 1. Introduction

Compounds exhibiting large magnetocaloric effect in the vicinity of room temperature have recently drawn much attention due to the possibility of their application in magnetic cooling devices. Among the different types of intermetallic compounds, two groups exhibiting magnetoelastic ferro to paramagnetic state transition are the most prospective. The first one, an La(Fe,Si)_13_ group of compounds, is being optimized by introducing cobalt and manganese into the composition and by performing hydrogenation [1,2,3,4,5,6]. All of these procedures are aimed toward shifting the Curie point to the desired room temperature. The second group, which is the subject of this study, constitutes a big family of Fe_2_P-based compounds. The three main groups are: (Mn,Fe)_2_(P,As), (MnFe)_2_(P,Ge) and (MnFe)_2_(P,Si) compounds. Our study is focused on the (Mn,Fe)_2_(P,As) group, which is known to exhibit the best magnetocaloric properties (highest adiabatic temperature change) [7,8,9,10,11,12,13]. Most of the studies have concentrated on intermetallic compounds based on four elements. In our study, we will investigate five-element intermetallic compounds with the overall formula: MnFeP_0.5_As_0.5−*x*_Ge*_x_*. In 2005, Tegus et al. published results of a germanium substitution for arsenic for a Mn_1.1_Fe_0.9_P_0.7_As_0.3−*x*_Ge*_x_* series [14]. In the studied samples, a composition with the germanium amount *x* = 0.1 was found to have the highest magnetic entropy change in the high magnetic field B = 3 T. Under a moderate magnetic field of B = 1 T, the highest magnetic entropy change was found for the composition with *x* = 0.15 (approximately −ΔS_M_ = 11 J^.^kg^−1.^K^−1^), however, adiabatic temperature change was not evaluated during this study. The Curie point for the composition with *x* = 0.10 was obtained as T_C_ = 270 K, while for *x* = 0.15 it was T_C_ = 290 K.

In our recent study, it was found that silicon as a fifth element (substituted for arsenic) may increase magnetic entropy change as well as adiabatic temperature change in Fe_2_P-type compounds. It was also found that the maximum adiabatic temperature change in silicon-doped MnFeP_0.35_As_0.65_ compounds can be correlated with the maximum crystal unit cell volume [15]. 

The main aim of this work is to study the impact of doping the known composition Mn_1.1_Fe_0.9_P_0.5_As_0.5_ by germanium Ge on structural and magnetocaloric parameters (adiabatic temperature change ΔT_ad_ and magnetic entropy change ΔS_M_). The second aim is related to finding correlations between structural changes and magnetocaloric properties. In this case, contrary to the silicon atom (atomic radius *r* = 110 pm), germanium has a higher atomic radius than arsenic (125 versus 115 pm), which should cause unit cell enlargement. Newly synthetized compounds will be studied by means of calorimetry, X-ray diffraction, microanalysis, magnetometry and magnetocalorimetry. Moreover, magnetic entropy change (ΔS_M_) under a moderate magnetic field (B = 1 T) will be calculated from magnetometric measurements. 

## 2. Materials and Method

Approximately 15 g samples were prepared by initial high energy ball milling the stoichiometric mixture of elemental powder Fe (3 N), red phosphorous P (2N5), elemental shots Mn (4N), Ge (5N) and FeAs_2_ (2N5) compounds under an argon atmosphere. Powders were then pressed in order to form pellets. Pellets were sintered for 5 h at 1273 K and homogenized for the next 10 h at 923 K under a vacuum 5 × 10^−5^ mbar (wire vacuum furnace, Czylok, Poland). Thereafter, samples were slowly cooled down with the furnace to room temperature.

The phase purity and the crystal structures were determined by powder X-ray diffraction (XRD) using Cu*K_α_* radiation (MiniFlex, Rigaku, Japan). The Rietveld refinement analysis of the X-ray diffraction data were performed using FullProf Suite Program 3.00 [16]. The microstructure observations and chemical composition evaluation was done by JCXA 733 (JEOL, Japan) equipped with energy dispersive (EDS) and wavelength dispersive (WDS) microanalyzers. The direct adiabatic temperature changes ΔT_ad_(T,B) were obtained with an adiabatic magnetocalorimeter (AMT&C Group, Russia) in a temperature range of 170–350 K under magnetic fields up to 1.7 T. The external magnetic field was changed in a cycle of 0T–1.7T–0T with a rate of 2 T/s, during ΔT_ad_ measurements. ΔT_ad_ measurements were performed on the samples that were initially milled, mixed and consolidated with 5% of polyvinylidene fluoride (PVDF) powder by heating to 573 K and pressing. Tablets prepared by this method are resistant to the large magnetovolume effect during the measurements, but exhibit lower maximum ΔT_ad_ than the bulk samples. Differential scanning calorimetry (DSC) studies were performed with use of a Pegasus 404c thermal analyzer (Netzsch, Germany). Samples with a mass of approximately 30 mg were heated with at the rate of 10 K/min under a helium atmosphere a gas flow equal to 50 mL/min.

Magnetic measurements were performed using a Physical Property Measurement System (PPMS-7, Quantum Design, Inc., San Diego, CA, USA). Compounds exhibiting first order magnetic transition were being studied. Therefore, it was advisable to perform isofield measurements or to use the so called “loop process” during isothermal measurements in order to avoid artifacts (“spikes”) at the calculations of magnetic entropy change stage. In this work, isofield measurements were carried out. Magnetization curves M = *f*(T), measured for different values of magnetic field induction were further interpolated to obtain the same temperature steps for every value of magnetic field induction, enabling use of the Maxwell relations-based formula for magnetic entropy change calculations [17]:

(1) $$\Delta S\;=\;\underset{i}{\sum }\frac{M\left(T+\frac{\Delta T}{2},B_{i}\right)-M\left(T-\frac{\Delta T}{2},B_{i}\right)}{\Delta T}\cdot \Delta B_{i}.$$

The magnetization was measured in a temperature range of 250–400 K under magnetic fields up to 1 T.

## 3. Results and Discussion

**Figure 1:**
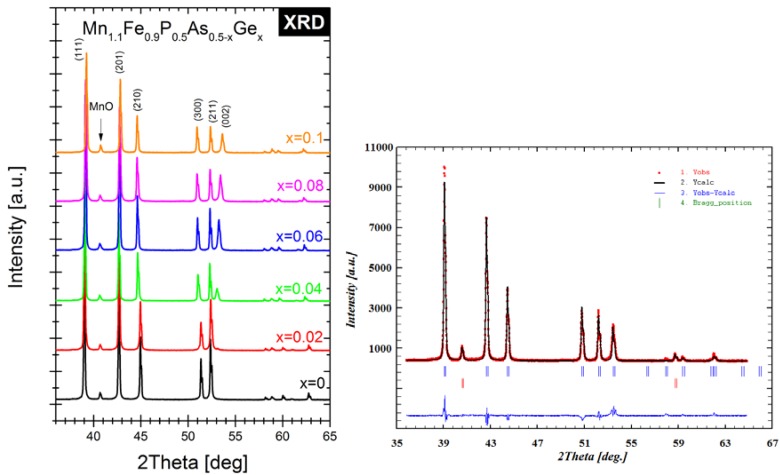
Diffraction patterns of all studied samples (**left**); Rietveld refinement plot for sample Mn_1.1_Fe_0.9_P_0.5_As_0.4_Ge_0.1_ (**right**).

**Figure 2:**
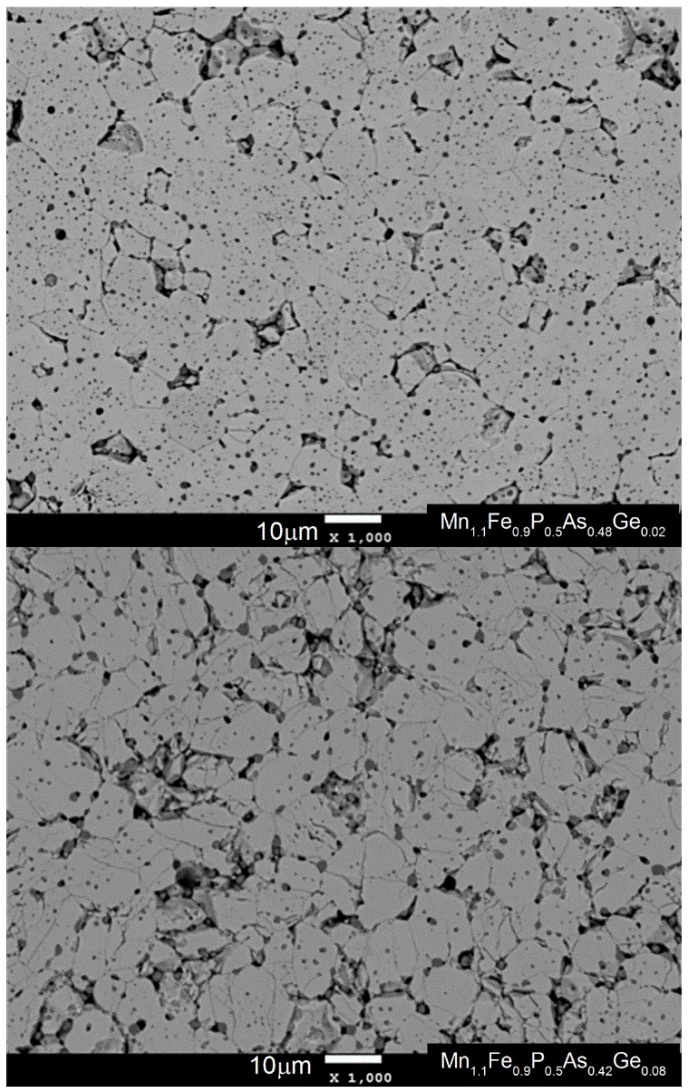
Microstructure of two different samples with germanium amounts of *x* = 0.02 and *x* = 0.08. At the measurement temperature, sample *x* = 0.02 is paramagnetic while *x* = 0.08 ferromagnetic.

Synthetized compounds were investigated by different experimental methods. The structure of polycrystalline samples was evaluated by XRD. In Figure 1 (left), one can see the diffraction patterns of all of the studied samples. It can be easily seen that all samples consisted of two phases i.e., major hexagonal Fe_2_P-type phase (space group number: 189, Hermann-Mauguin symbol: P −6 2 m) and MnO impurity (space group number: 225, Hermann-Mauguin symbol: F m −3 m). By analyzing the diffraction patterns from 50 to 55 degrees in the 2θ range, it is easily seen that position of the (002) diffraction peak shifts into the smaller value in the 2Theta scale into the position of the (211) peak. Thus, in a high temperature phase (paramagnetic) three maxima are visible instead of two. For paramagnetic samples, the (002) peak shifts into the higher value of the 2Theta value and the so-called “diffraction triplet” from the (300), (211) and (002) peaks is visible in the 50–55 degrees region. Therefore, the sample with composition MnFeP_0.5_As_0.48_Ge_0.02_ is ferromagnetic (FM) at room temperature, while MnFeP_0.5_As_0.46_Ge_0.04_ is paramagnetic (PM). Both compounds exhibit FM-PM transition near room temperature. The weight fractions of the phases, lattice parameters, unit-cell volume and *c*/*a* ratio for the Fe_2_P-type phase were then refined using the Rietveld method, and are presented in Table 1. The selected Rietveld refinement pattern for the sample of Mn_1.1_Fe_0.9_P_0.5_As_0.4_Ge_0.1_ is shown in Figure 1 (right). The purity of the synthetized compounds was checked by performing microstructural analysis. In Figure 2, SEM images of two samples with germanium amounts of *x* = 0.02 and *x* = 0.08 have been presented. The system with the amount of germanium *x* = 0.02 is ferromagnetic at room temperature, while the sample with *x* = 0.08 is paramagnetic. The average as well as phase composition was evaluated by means of EDS and WDS analysis. It was found that the paramagnetic sample is more porous than the ferromagnetic sample. Performed microanalysis showed that the manganese oxide MnO can be found mainly in the pores of the presented structure.

Nominal and measured compositions of two samples (*x* = 0.02, *x* = 0.08) are shown in Table 2. 

The nominal composition stays in good agreement with the measured one. All of the samples have a disturbed Mn/Fe ratio because of MnO second phase formation. From the WDS measurement of the chemical compositions, the amount of MnO was examined. It is equal to 4 at % in the case of the sample with *x* = 0.02 and 5 at % in the case of the second sample (*x* = 0.08). 

**Figure 3:**
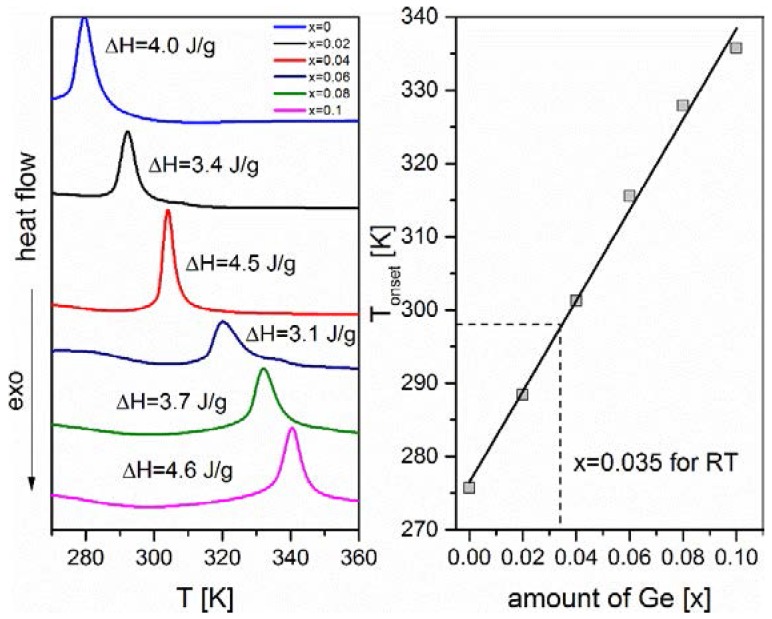
Results of DSC measurements. In the left panel, heating curves (10 K/min) for all studied compounds are presented. Structural transition enthalpy was evaluated. In the right panel, onset temperatures of structural transition versus reciprocal temperature are plotted. From the linear fit, the transition at RT was found for composition *x* = 0.035.

A series of studied compounds exhibit magnetoelastic first-order transition. DSC was used to evaluate latent heat of transition in the absence of a magnetic field. In Figure 3, one can see the thermograms for all of studied samples up to a germanium amount of *x* = 0.1. The latent heat changes in the range of 3.1–4.6 J/g, and is lowest for the sample with *x* = 0.06. In the range of compositions from *x* = 0.04 to *x* = 0.10, the minimum transition enthalpy is can be easily noted at *x* = 0.06. It was also found that slight amount of germanium (*x* = 0.02) caused a decrease of latent heat from 4.0 to 3.4 J/g. The temperature of transition depends linearly on the germanium amount in the sample. It varies from 276 K for the pure sample to 336 K for the germanium-doped sample with *x* = 0.10. It can be also be predicted from the linear fit that the transformation at room temperature occurs for the composition with *x* = 0.035. 

**Figure 4:**
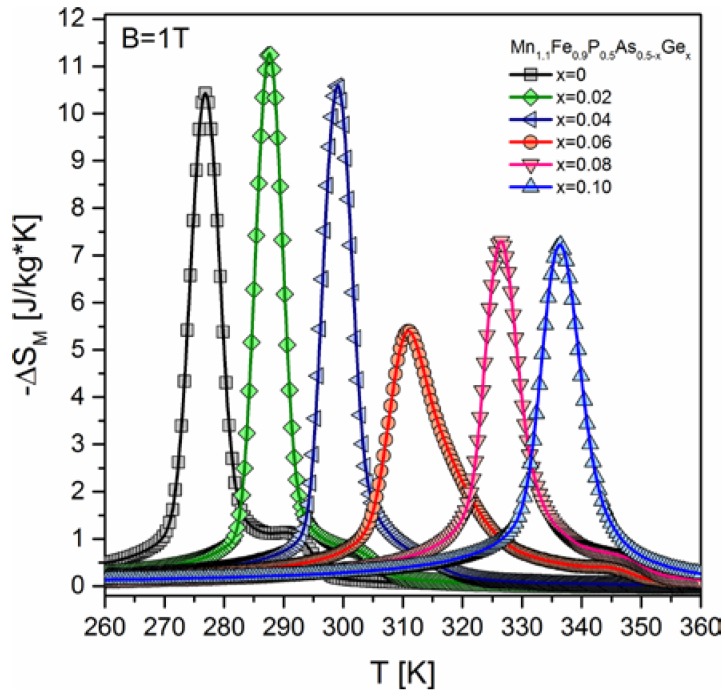
Magnetic entropy change calculated for all of the studied samples for magnetic field induction B = 1 T.

Magnetic entropy change was calculated for all of the studied samples for magnetic field change up to B = 1 T. As one can see in Figure 4, the magnetic entropy change (at B = 1 T) drops significantly with increasing concentration of germanium from *x* = 0.04 to *x* = 0.06, where it achieves a minimal value (−ΔS_M_ = 5.5 J^.^kg^−1.^K^−1^). 

**Figure 5:**
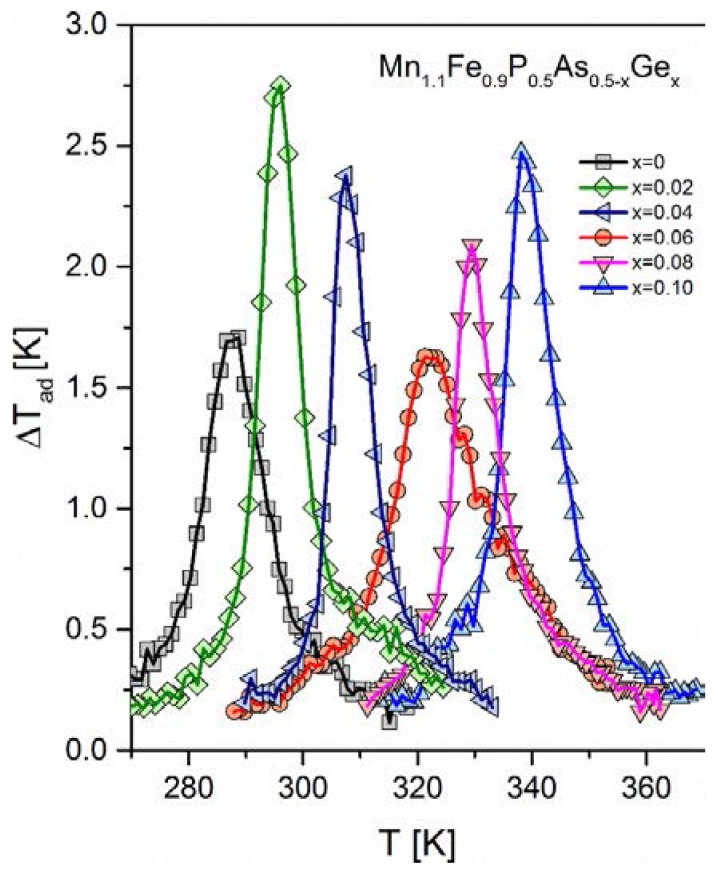
Adiabatic temperature change of all of the studied samples measured in a cooling regime under a magnetic field of B = 1.7 T.

The highest magnetic entropy change was obtained for a sample with the germanium amount of *x* = 0.02 (−ΔS_M_ = 11.0 J^.^kg^−1.^K^−1^). Adiabatic temperature change (see Figure 5 for details) behaves the same way as the magnetic entropy change. It was found that by the slight substitution of germanium for arsenic (*x* = 0.02), the adiabatic temperature change raised from 1.75 to 2.75 K at B = 1.7 T. This quantity exhibits similarly as the magnetic entropy change minimum at the germanium amount of *x* = 0.04. For this composition, the adiabatic temperature change equals 1.7 K. It can be easily noticed that germanium substitution for arsenic leads to an increase of the Curie point. The sample exhibiting the highest magnetocaloric effect (*x* = 0.02) exhibits FM-PM transition at room temperature, which is advantageous from a practical point view.

**Figure 6:**
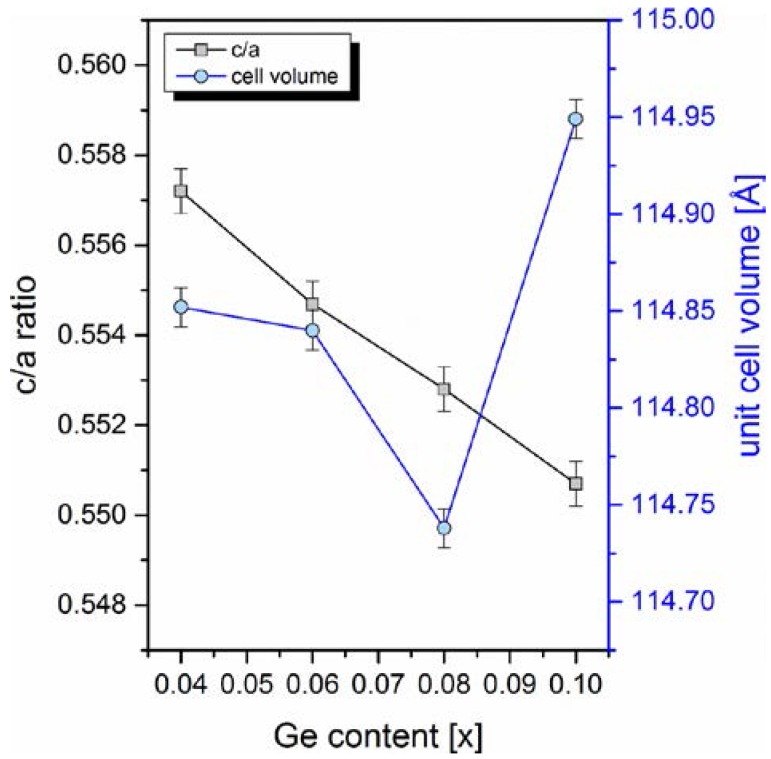
Unit cell volume and lattice parameters ratio (*c*/*a*) as a function of germanium content, derived from XRD measurements (for ferromagnetic samples at T = 298 K). Lines are guides for the eyes.

**Figure 7:**
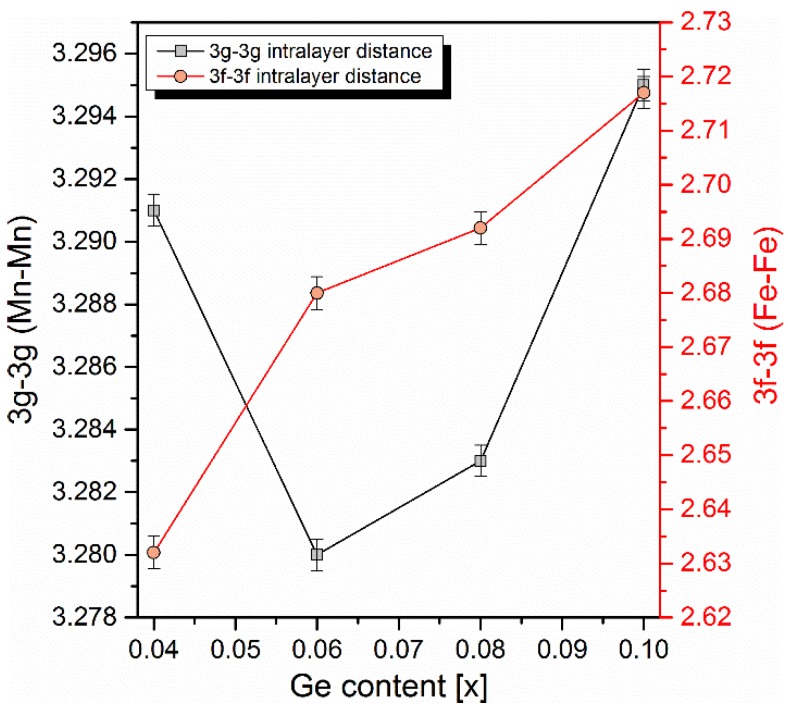
Intralayer *3g*-*3g* and *3f*-*3f* atom distances as a function of germanium content (data presented for ferromagnetic samples at T=298K). Lines are guides for the eyes.

Generally, the magnetocaloric effect is related to both the magnetic entropy change and latent heat of transition. In the MnFePAsGe series, the adiabatic temperature change has a similar germanium content dependence as the magnetic entropy change. Therefore, it can be assumed that magnetocaloric effect is mainly altered due to the change of the overall magnetic moment of the unit cell. Many studies, up to now, have indicated that the highest magnetic moment is localized mainly on manganese atoms (~2.9 μB) which prefer the *3g* Wyckoff position of the hexagonal P −6 2 m lattice cell. On the other hand, iron atoms prefer the *3f* positions (magnetic moment approximately 1.3 μB) and, in consequence, both elements form alternating planes (layers) in the crystal. Therefore, it can be predicted that mainly the modification of *3g-3g* intralayer distances leads to large fluctuations of the cell’s magnetic moment and consequently to the alteration of magnetocaloric effect. Despite the fact that germanium atoms have a higher atomic radius than arsenic (125 pm for Ge versus 115 pm for As), a substitution of germanium for arsenic up to the value of *x* = 0.08 leads to the unit cell volume contraction. Moreover, the ratio of lattice constants *c*/*a* decreases with the increasing amount of germanium in the whole range of studied germanium concentrations. Both parameters have been refined from the X-ray diffraction data, and are gathered in Figure 6. Such modifications of structure leads to the change of magnetic atoms (Fe and Mn) distances in and between layers. It was found that the composition-dependent magnetocaloric parameters can be correlated with the changing *3g-3g* intralayer distances. In Figure 7, *3g-3g* and *3f-3f* distances are plotted as a function of germanium content. As one can see, the *3g-3g* distance exhibits the same minimum as was found for adiabatic temperature and magnetic entropy change. Contrary to the behavior of the *3g-3g* distance, the *3f-3f* intralayer distance rises in the whole range of germanium concentrations. Assuming that Mn atoms are placed in the *3g* Wyckoff positons, by substituting a non-magnetic metalloid atom, i.e., arsenic or phosphorous, for atoms with different atomic radius, one can control the Mn-Mn atomic distance in a crystalline cell, which affects magnetocaloric effect. The sample with composition Mn_1.1_Fe_0.9_P_0.5_As_0.48_Ge_0.02_ has certain parameters, i.e., a magnetic entropy change calculated for magnetic field induction of B = 1 T and the Curie temperature, similar to the sample obtained by Tegus et al. with composition Mn_1.1_Fe_0.9_ P_0.7_As_0.2_Ge_0.1_. 

## 4. Summary

It was found that the substitution of the non-magnetic site in (Mn,Fe)_2_(P,As) quaternary compounds by the fifth element may lead to greatly enhanced magnetocaloric properties induced by the alteration of distances of magnetic elements (Mn,Fe). Germanium-doped quinary Mn_1.1_Fe_0.9_P_0.5_As_0.5−*x*_Ge*_x_* compounds were obtained as an almost pure magnetocaloric phase with a small amount of MnO impurity. Germanium doping at *x* = 0.02 raised the adiabatic temperature change from 1.75 to 2.75 K at B = 1.7 T, i.e., by 40%, while the magnetic entropy change at B = 1 T was raised by 10% (from −10.5 to −11.5 J^.^kg^−1.^K^−1^). For the germanium fraction *x* = 0.06, the magnetocaloric effect is at the minimum (ΔT = 1.6 K, ΔS = −5.5 J^.^kg^−1.^K^−1^), which corresponds to the minimal value of the *3g-3g* intralayer distance (position of manganese atoms). 

## Figures and Tables

**Table 1:** The weight fractions of the refined phases, room temperature lattice parameters, calculated *c/a* ratio and unit-cell volume for the Fe_2_P-type phase.

Ge Content:	Fe_2_P-type (%)	MnO (%)	a = b (Å)	c (Å)	*c*/*a* Ratio	V (Å^3^)
*x* = 0	95.01 ± 1.50	4.99 ± 0.29	6.1716 ± 0.0003	3.4977 ± 0.0001	0.5667	115.377 ± 0.008
*x* = 0.02	95.29 ± 0.70	4.71 ± 0.27	6.1679 ± 0.0002	3.4932 ± 0.0001	0.5664	115.089 ± 0.007
*x* = 0.04	94.02 ± 0.75	5.98 ± 0.35	6.1972 ± 0.0002	3.4431 ± 0.0001	0.5572	114.852 ± 0.007
*x* = 0.06	94.13 ± 1.30	5.87 ± 0.40	6.2060 ± 0.0004	3.443 ± 0.0002	0.5547	114.840 ± 0.012
*x* = 0.08	94.97 ± 1.23	5.03 ± 0.32	6.2117 ± 0.0003	3.4337 ± 0.0002	0.5528	114.738 ± 0.010
*x* = 0.1	93.67 ± 0.53	6.33 ± 0.38	6.2232 ± 0.0004	3.4273 ± 0.0002	0.5507	114.949 ± 0.012

**Table 2:** Comparison of nominal and measured compositions of two whole samples (main phase + MnO impurity) and major magnetocaloric phase.

	Sample 1 *x* = 0.02	Sample 2 *x* = 0.08
Nominal composition	Mn_1.10_Fe_0.90_P_0.50_As_0.48_Ge_0.02_	Mn_1.10_Fe_0.90_P_0.50_As_0.42_Ge_0.08_
WDS average	Mn_1.12_Fe_0.91_P_0.47_As_0.48_Ge_0.02_O_0.13_	Mn_1.13_Fe_0.90_P_0.49_As_0.41_Ge_0.07_O_0.17_
WDS main phase	Mn_1.07_Fe_0.92_P_0.48_As_0.49_Ge_0.02_	Mn_1.06_Fe_0.95_P_0.49_As_0.42_Ge_0.08_
EDS average	Mn_1.12_Fe_0.91_P_0.47_As_0.48_Ge_0.02_O_0.23_	Mn_1.14_Fe_0.93_P_0.48_As_0.38_Ge_0.06_O_0.19_
